# Comparison of the effect of recombinant human pro-urokinase and tirofiban on myocardial blood flow perfusion in ST elevation myocardial infarction patients receiving primary percutaneous coronary intervention: A one-center retrospective observational study

**DOI:** 10.1097/MD.0000000000016143

**Published:** 2019-07-05

**Authors:** Zhuhua Yao, Wenting Li, Lisong Cheng, Mingying Cao, Zhihua Pang, Yongbin Li

**Affiliations:** Department of Cardiology, Tianjin Union Medical Center, Tianjin, China.

**Keywords:** primary percutaneous coronary intervention, recombinant human pro-urokinase, retrospective study, ST elevation myocardial infarction, tirofiban

## Abstract

Ischemia/reperfusion (I/R) injury is associated with primary percutaneous coronary intervention (PPCI). The current study was performed to compare the effect of tirofiban and recombinant human pro-urokinase (rh-proUK) on the improvement of coronary slow blood after PPCI.

Sixty-five ST elevation myocardial infarction (STEMI) patients treated with rh-proUK and an equal number treated with tirofiban after PPCI were employed in the current study. The clinicopathological information regarding the biochemical parameters, thrombolysis in myocardial infarction (TIMI) grade, hemodynamics parameters, thrombus core (TS), sum-STR, left ventricular ejection fraction (LVEF), blood routine parameters, high-sensitivity C-reactive protein (CRP) level, uric acid, hepatorenal function, electrocardiogram (ECG), and echocardiography before and after the interventions were collected. The differences in those parameters between the 2 groups then compared with assess the treatment effect and side effects associated with the both therapies.

The results showed that the TIMI level post-intervention (*P* = .03), the proportion of TIMI myocardial perfusion grade level III (*P* = .04), the changes in thrombus score (*P* < .001) in rh-proUK group were significantly higher than those in tirofiban group while the corrected TIMI Frame Count (CTFC) (*P* = .02), the incidence of slow flow (*P* = .02), the thrombus score post-intervention (*P* < .001), the stent length (*P* = .02), and the number of receiving administration of sodium nitroprusside (*P* = .01) were significantly lower than those in tirofiban group. Moreover, the levels of CK (*P* < .001), CK-MB (*P* = .01), and NT-proBNP 24-hour post-intervention (*P* < .02) were significantly lower in rh-proUK group than those in tirofiban group and the sum-STR right after the intervention (*P* < .03) of rh-proUK group was significantly higher than that of tirofiban group. No significant difference was detected between the 2 therapies regarding major adverse cardiac events (MACE).

The findings outlined in the current study showed that the improvement effect of rh-proUK on blood flow condition was stronger right after the intervention and the therapy had a similar safety when compared with tirofiban during a 30-day follow-up.

## 1 Introduction

Characterized by the sudden attack and the high mortality, ST elevation myocardial infarction (STEMI) has been conceived as one of the major death-related factors in developing countries.^[1]^ Generally, the onset of STEMI is resulted from the progression of coronary atherosclerosis. The dysfunction of endothelial tissues during atherosclerosis contributes to the initiation of inflammation in vessels, which leads to the fracture of atherosclerotic plaques and ulcer.^[2]^ Subsequently, platelets are activated due to the endothelial dysfunction and induces the formation of thrombus as well as the obstruction of coronary arteries.^[3]^ Primary percutaneous coronary intervention (PPCI) is the mostly adopted strategy to handle STEMI.^[3]^ The intervention has the advantage to re-establish blood flow of the obstructed sites in a relatively short period and has been proposed as the first-line therapy for coronary ischemia.^[4]^ However, PPCI can itself lead to reperfusion injuries characterized by coronary slow flow/no flow, which causes secondary damages to patients.^[5]^ Thus, how to attenuate side effects associated with the application of PPCI has become a hot subject in the clinical treatment of STEMI.

Coronary slow flow/no flow is termed as the absent of blood flow in vessels even after the treatment of PPCI or thrombolysis.^[5]^ The mechanism driving the formation of slow flow/no flow is complicated and one the of the major factor contributing to the symptom is the aggregation of small thrombus in coronaries induced by the adhesion of platelets.^[6]^ The key role platelet in the coronary micro-cycle implies that the targeted regulation of the platelet amount may represent a promising strategy for the management of coronary slow flow/no flow after the treatment of PPCI.

The aggregation of platelet is driven by the interaction between surface specific glycoprotein antigens IIb/IIIa.^[7]^ Therefore, it is well recognized that the application of antagonists of specific glycoprotein antigen can play an anti-platelet function via the inhibition of IIb/IIIa.^[7]^ As the only clinically available specific antagonist of IIb/IIIa, tirofiban has the advantages of short biological half-life and high specificity.^[8]^ The administration can block thrombus formation and attenuate coronary vessel obstruction by inhibiting the aggregation of platelets,^[8]^ which ameliorates coronary micro-cycle conditions. Except for the application of anti-platelet drugs, the utilization of recombinant human pro-urokinase (rh-proUK) is also being gradually recommended for the management of slow flow in recent years.^[9]^ Previous study demonstrated that the load of infarction related artery (IRA) in STEMI patients was relatively high, which was a major risk factor for coronary slow flow/no flow after PPCI treatment. However, the injection of rh-proUK can dissolve small thrombus that contributes to the formation slow flow/no flow.^[10]^ Given the regulation effect of rh-proUK on coronary blood flow, rh-proUK also has the potential to handle slow flow/no flow associated with PPCI administration. Nevertheless, compared with the studies focusing on the treatment outcomes of tirofiban, very few studies have paid attention to the treatment effect of rh-proUK on coronary micro-cycle after PPCI. Thus, the comparison of the treatment outcomes on PPCI-induced coronary slow flow/no flow between the 2 therapies might provide novel information on the clinical improvement of PPCI.

To elucidate the differences between the 2 therapies on coronary micro-cycle after PPCI, the treatment outcomes of 65 STEMI patients receiving PPCI and tirofiban treatment and an equal number of patients treated with rh-proUK were collected in the current study. Then the effect of tirofiban and rh-proUK on the biochemical parameters, sum of ST-segment resolution (sum-STR), hemodynamics parameters, and major adverse cardiac events (MACE) was compared with demonstrates which therapy might have better effect in improving coronary micro-cycle in STEMI patients receiving PPCI.

## 2 Methods

### 2.1 Patients

The study was a retrospective study using case information and patients employed in the current study were selected based on the following criteria: STEMI symptoms were diagnosed based on Guidelines for the diagnosis and treatments of STEMI published by Chinese Society of Cardiology of Chinese Medical Association^[11]^; patients received PPCI treatments within 12 hours after the admissions; patients were treated with tirofiban or rh-proUK after PPCI; patients had no anaphylactic reaction after tirofiban or rh-proUK treatments; the patients received no intravenous thrombolysis treatments before PPCI; patients with Killip level higher than III or cardiac shock. Based on the above criteria, the history of information of 130 STEMI patients were collected in the current study from Oct 2016 to Nov 2017 in the Tianjin Union Medical Center. Among the cohort, 65 cases were treated with tirofiban and the left cases were treated with rh-proUK. All the patients possessed detailed clinicopathologic features and signed Informed Consents for the utilization of the information. The study was approved by the ethic committee of the Tianjin Union Medical Center for the data collection of the patients (Approval NO: MEC-2016–06). All the works were undertaken following the provisions of the Declaration of Helsinki.

### 2.2 Treatment strategy

All the patients were orally administrated with 300 mg aspirin and 300 mg clopidogrel/180 mg ticagrelor, and intravenously injected with 5000 IU heparin upon admission. Coronary angiogram (CAG) was performed to determine the number of pathological branches before the interventions. For patients treated with rh-proUK, 5 mg drug was intravascularly injected during the intervention and another administration of 5 mg rh-proUK was given to the patients after the insertion of the stent. If the symptoms of slow flow still exited, the patient was administrated with 5 mg rh-proUK for the last time. For patients treated with tirofiban, 5 μg/kg was slowly injected via coronary arteries during the intervention. After the insertion of stent, another administration of 5 μg/kg tirofiban was given to the patients via coronary arteries. For patients still showed symptoms of slow flow, 3 μg/kg tirofiban was injected via coronary arteries. Twenty four hours after the intervention, all the patients were administrated with 100 mg aspirin and 75 mg clopidogrel/90 mg ticagrelor as well as 5000 IU heparin for 5 to 7 days. All the patients were followed up for 30 days after the discharge to record MACE.

### 2.3 Treatment outcome measurement

To compare the treatment effect and side effects associated with the 2 therapies, the information regarding TIMI grade, hemodynamics parameters, thrombus core (TS),^[12]^ sum-STR, left ventricular ejection fraction (LVEF), the blood routine parameters, high-sensitivity C-reactive protein (CRP) level, uric acid, hepatorenal function, electrocardiogram (ECG), and echocardiography were collected both before and after the interventions, and compared with determine the differences in the treatment effect and side effects of the 2 therapies.

### 2.4 Statistical analysis

Statistical analyses were conducted using SPSS version 19.0 software (IBM, Armonk, NY). Continuous data were expressed as the mean ± standard deviation (SD). Categorical data were represented by frequency distribution or case number. The Student *t* test was used to compare the difference between groups of continuous data. Chi-square and Fisher exact tests were used to determine if differences existed between different groups of categorical indices. *P* < .05 was considered as statistical significance.

## 3 Results

### 3.1 Baseline information

The analyses of the baseline information between the 2 groups showed that no significant difference was detected regarding age, sex, body mass index (BMI), Killip score, smoking history, drinking history, and other indicators before the surgeries. The detailed information was shown in Table 1. Moreover, no significant difference was detected regarding the combined application of drugs between the 2 groups (Table 2).

**Table 1:**
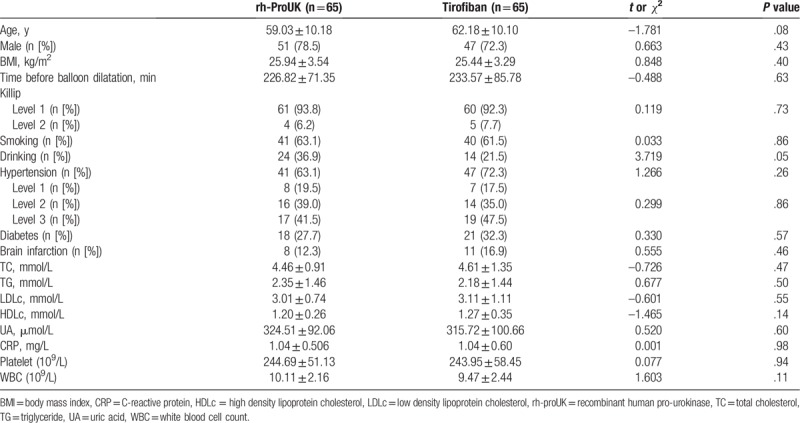
Baseline information of the patients.

**Table 2:**
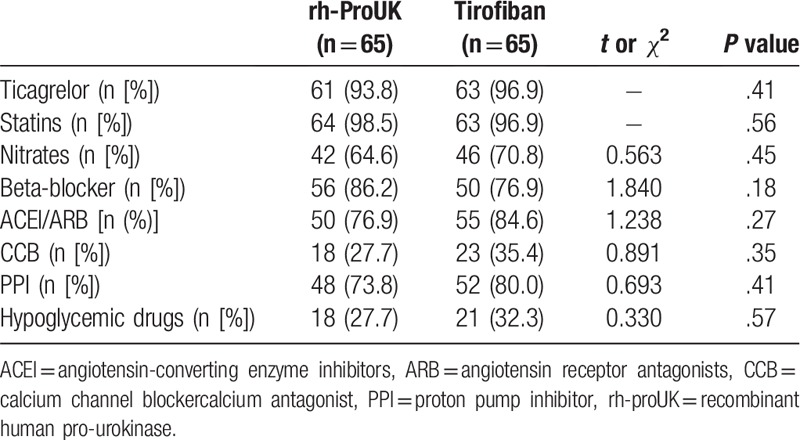
Information regarding the combined use of drugs.

### 3.2 Intervention information

As shown in Table 3, no significant difference was detected regarding the branch number, the TIMI level before the intervention, the stent number, the proportion of thrombus aspiration, the thrombus score prior-intervention, the proportion of hypotension during the intervention, the systolic pressure, and the diastolic blood pressure. However, the TIMI level post-intervention, the proportion of TIMI myocardial perfusion grade level 3, the changes in thrombus score of rh-proUK group were significantly higher than those in tirofiban group (Table 3). To the contrary, the corrected TIMI Frame Count (CTFC), the incidence of slow flow, the thrombus score post-intervention, the stent length, and the number of receiving administration of sodium nitroprusside were significantly lower than those in tirofiban group (Table 3).

**Table 3:**
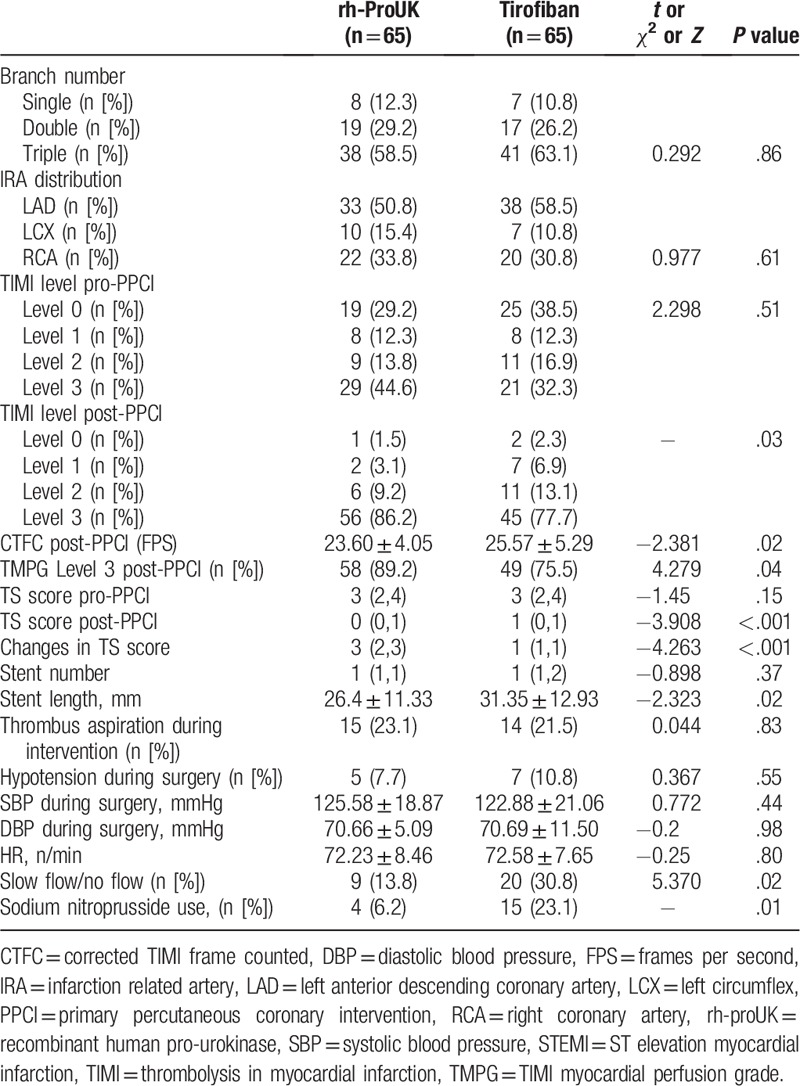
Information regarding STEMI and PPCI treatment outcome of the patients.

### 3.3 Effect of rh-proUK or tirofiban on treatment outcomes of PPCI

**Figure 1:**
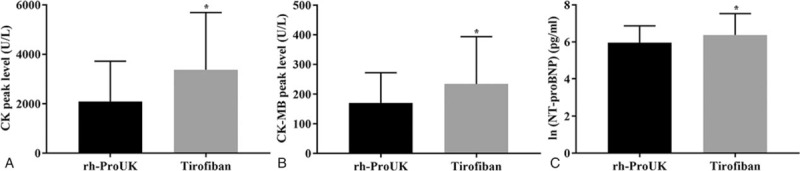
Effect of the 2 therapies on the biochemical parameters of the patients. A, Quantitative analysis results of CK peak level. B, Quantitative analysis results of CK-MB peak level. C, Quantitative analysis results of ln(NT-proBNP) level. “^∗^” *P* < .05 versus rh-ProUK group.

**Figure 2:**
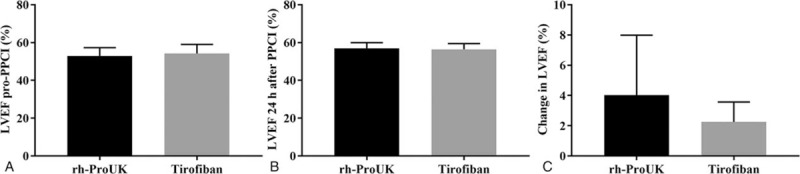
Effect of the 2 therapies on the sum-STR 2 hours after PPCI surgery. A, Quantitative analysis results of LVEF pro-PPCI level. B, Quantitative analysis results LVEF 24 hours after PPCI. C, Quantitative analysis results of change in LVEF. LVEF = left ventricular ejection fraction, PPCI = primary percutaneous coronary intervention.

After PPCI, the levels of CK, CK-MB, and NT-proBNP 24-hour post-intervention were significantly lower in proUK group than those in tirofiban group (Fig. 1). Nevertheless, regarding the time point for recording the peak levels of CK (*P* = .13) and CK-MB (*P* = .18), no significant difference was detected between the 2 groups. The sum-STR right after the intervention of rh-proUK group was also significantly higher than that of tirofiban group (Table 4) while sum-STR 2-hour post-intervention showed no significant difference (Table 4). Additionally, the changes in LEVF in rh-proUK group were significantly higher than that in tirofiban group (Fig. 2).

**Table 4:**
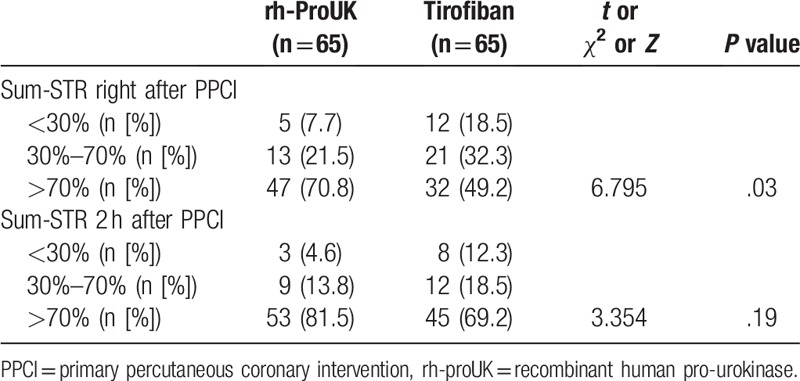
Effect of the 2 therapies on the sum-STR 2 hours after PPCI surgery.

### 3.4 MACE

The incidences of angina pectoris and heart failure in rh-proUK group were significantly lower than those in tirofiban group (Table 5). To the contrary, the incidences of TIMI microbleed and global utilization of streptokinase and tissue plasminogen activator for occluded coronary arteries microbleed, including urine occult blood, fecal occult blood, epistaxis, and gingival bleeding, in rh-proUK group were significantly higher than those in tirofiban group (Table 5). No intracranial hemorrhage, myocardial infarction, or other severe side effects were detected in the 2 groups (Table 5).

**Table 5:**
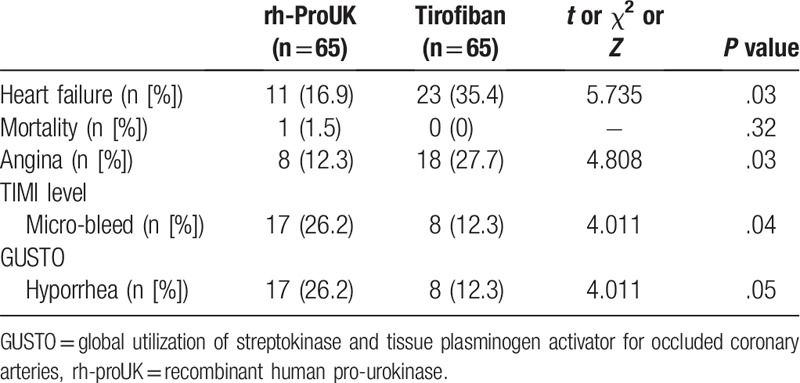
Effect of the 2 therapies on the MCAE in the patients.

### 3.5 Follow-up

The patients were followed up for 30 days after the discharge from the hospital and no loss to follow-up was recorded. Based on the information shown in Table 6, no significant differences regarding the physiological indices and MACE were detected between the 2 groups (Table 6).

**Table 6:**
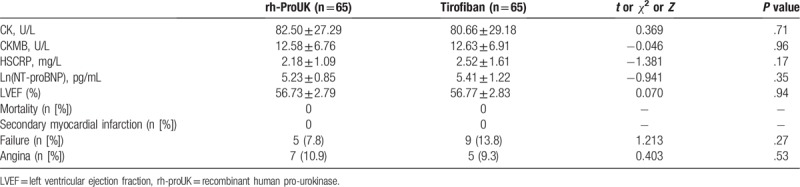
Information regarding prognosis in a 30-day follow-up.

## 4 Discussion

PPCI can effectively dredge the infarcted coronary arteries and has been selected as the first-line treatment strategy for the clinical management of STEMI.^[13]^ The recent investigation based on large samples inferred that PPCI could establish blood flow in >90% IRA and restore TIMI to level 3, which reduced the mortality rate of acute myocardial infarction (AMI) to as low as 3% to 4%.^[13]^ However, the efficacy of PPCI is counteracted with some severe side effects such as slow flow/no flow induced by I/R injuries. Slow flow/no flow is one of the risk factors influencing the prognosis of STEMI patients. To improve the treatment outcomes of PPCI, some agents such as tirofiban and rh-proUK are being applied to ameliorate the coronary blood flow after PPCI.

Compared with the studies based on tirofiban, few attention has been paid to the effect of rh-proUK on coronary blood flow after PPCI. In the study of Guo et al,^[9]^ the author showed that the application of rh-proUK significantly lowered the slow blood incidence when compared with patients solely treated with PPCI, indicating that the application of rh-proUK had the potential to ameliorate blood flow condition after PPCI. Thus, in the current study, we compared the treatment effect of rh-proUK and tirofiban on slow flow/no flow associated with PPCI. The results showed that the application of rh-proUK significantly decreased the incidence of slow flow after PPCI when compared with tirofiban group. Moreover, the thrombus score in patients treated with rh-proUK was also significantly lower than that in tirofiban group, which might be resulted from the stronger thrombolysis effect of rh-proUK against thrombus already existing in coronaries.^[10]^ Regarding biochemical parameters, the peak levels of CK and CK-MB in rh-proUK group were significantly lower than those in tirofiban group while the peak time point showed no significant difference between the 2 groups. Both indicators are well-recognized indicators for myocardial function in clinic.^[11]^ Moreover, the level of NT-proBNP which represents the myocardial damage degree^[14]^ was also lower in proUK group than that in tirofiban group. Taken together, the data evidently indicated the stronger protection effect of rh-proUK on injuries associated with PPCI treatment than tirofiban.

The myocardial function of the patients was also evaluated with ECG and it was found that the change in sum-STR in rh-proUK group was larger than that of tirofiban group. Previous study showed that the recovery of myocardial ischemia injuries was in positive relation to the restoration of sum-STR.^[15]^ Combined with the larger change in LVEF in rh-proUK group, it was evident that rh-proUK could also improve the myocardial function much stronger than that of tirofiban. Additionally, the incidence of MACE occurring in rh-proUK group was also lower than that in tirofiban group, the results were representative of the safety of the application of rh-proUK in clinic. The current study also performed a 30-day follow-up after all the patients discharged from the hospital and the results showed that no significant difference was detected regarding parameters of biochemistry, myocardial function, and MACE between the 2 groups, further confirming the promising application potential of rh-proUK in ameliorating coronary blood flow after PPCI. The current study also showed that the stronger effect of rh-proUK on heart was more frequently detected right after the intervention, but with the time lapsing, the difference in the treatment effect between the 2 drugs gradually descended, which might be due to the stronger effect of rh-proUK in improving micro-cycle by dissolving small clots instead of antagonizing the aggregation of platelets that were utilized by tirofiban.^[10]^

Collectively, the current study compared the effect of rh-proUK and tirofiban in protecting STEMI patients against ischemia/reperfusion (I/R) injuries associated with PPCI. Our results showed both drugs could effectively decrease the incidence of slow flow and MCAE as well as improving myocardial function. Moreover, the effect of rh-proUK was stronger in improving most indicators right after the intervention and showed similar safety in application when compared with tirofiban during a 30-day following up, which indicated the promising application potential of rh-proUK in clinical management of slow flow associated with PPCI. However, the sample size of the current study was still small and might result in the bias of some detections. Thus, more comprehensive work with larger sample size are needed in the future to facilitate the promotion of rh-proUK.

## Author contributions

**Data curation:** Zhuhua Yao, Wenting Li.

**Formal analysis:** Wenting Li, Lisong Cheng, Mingying Cao, Yongbin Li.

**Methodology:** Lisong Cheng, Mingying Cao, Yongbin Li.

**Supervision:** Zhuhua Yao.

**Writing – original draft:** Zhuhua Yao.

**Writing – review & editing:** Zhihua Pang.
